# Summer Complaint

**Published:** 1870-09

**Authors:** 


### SUMMER COMPLAINT.

This is the name given to a disease of which thousands die every summer. It is indicated by frequent thin passages from the bowels, which rapidly debilitate. In a very few hours, sometimes, the full-faced sprightly child looses its plumpness and vivacity, and becomes listless, languid and almost pallid as a corpse. This ailment is often connected with teething, but not necessarily, for teething is a natural and healthful process of nature. Generally, "summer complaint" arises from too frequent feeding of children; from excessive heat followed by cooling off too quickly, from exposure to draughts of air, damp clothing, and sleeping uncovered. But in three cases out of four, unwise feeding excites the disease. By attributing any disease to the wrong cause, it is multiplied in consequence of the real cause being allowed to be kept in operation unchecked. It is a very common thing to attribute summer complaint of children to the use of unripe and imperfect and decayed or old fruits and berries in a souring condition. But it is the children of the poor who perish, ten to one, over those of the rich. When early apples sell at three cents a piece, and cherries five cents for a small teacupful, and other fruits and berries in the same proportion, it is clearly impossible that these articles should be a cause of disease among the children of the poor. Three fourths of the fatal diseases of childhood would be prevented by judicious feeding, which means chiefly that they should be fed regularly, and not be allowed to eat between meals. Until three months old, a child should be fed every two hours; from three to eight months, every three hours; from eight to twelve months, every four hours, and therefore four times during daylight. After a child is six months old, it should be trained to be nursed or fed but once from the time the mother goes to bed until the time for rising in the morning; thus preventing the mother from being waked out of the sleep she so much needs, and for the want of which, half the mothers have a draggled, nearly half-dead look during the whole time of nursing.

After a child is three months old, it should not be allowed to sleep in the same bed with its mother, but in a crib at the bedside, where it should be put immediately after each nursing; both mother and child will sleep better for this arrangement. Sometimes children seem to be cured of summer complaint, especially if connected with teething, by being allowed to chew a piece of bacon well-cooked, that is, the fat part of it attached to the skin; they will sometimes chew upon this for half an hour or more, with apparent delight.

Benefit has been derived by cutting up raw beef fine, pounded, then put in a rag and squeezed out, the solid part remaining to be eaten, and nothing else, a teaspoonful four times a day; and drinking but very little of anything except flaxseed tea or barley water. These things are to be done only until you can get a physician. It is horrible to think of a parent groping along in the dark, on his or her own responsibility, when the life of a child is at stake.

The public ought to be advised that all "soothing syrups," and all preparations of opium, laudanum, paregoric, and the like, have a direct tendency in all cases of summer complaint in children, to abate the symptoms only to induce a protracted recovery, convulsions, or water on the brain.

